# Assessing the combined effects of household type and insecticide effectiveness for kala-azar vector control using indoor residual spraying: a case study from North Bihar, India

**DOI:** 10.1186/s13071-019-3670-x

**Published:** 2019-08-22

**Authors:** Rakesh Mandal, Vijay Kumar, Shreekant Kesari, Pradeep Das

**Affiliations:** Department of Vector Biology and Control, Rajendra Memorial Research Institute of Medical Sciences (ICMR), Agamkuan, Patna, 800 007 Bihar India

**Keywords:** Kala-azar, Sand fly control, Spatio-temporal distribution, Indoor residual spraying, Insecticide susceptibility, Residual efficacy, Risk-mapping

## Abstract

**Background:**

Indoor residual spraying (IRS) is the mainstay for vector control intervention of visceral leishmaniasis (VL) in India. Little is known on the control effects of IRS on different household types. Here, we assessed if IRS with insecticides has an equal residual and interventional effect on all household types in a village. We also developed a combined spatial-risk map and a sand fly, *Phlebotomus argentipes* density analytical model based on household characteristics, insecticide susceptibility and IRS-status to explore the spatio-temporal distributions of the vector at a micro-scale level.

**Methods:**

This study was carried out in two villages of Mahnar block in Vaishali district, Bihar. IRS using two insecticides [dichlorodiphenyltrichloroethane (DDT 50%) and synthetic pyrethroid (SP 5%)] was evaluated for VL-vector (*P. argentipes*) control. Temporal residual efficacy of the insecticides on different wall-surface types was evaluated using the cone-bioassay technique according to WHO guidelines. Insecticide susceptibility of local *P. argentipes* was explored using the tube-bioassay method. Pre- and post-IRS sand fly densities were monitored in human dwellings and animal shelters using Centers for Disease Control light-traps installed between 18:00–6:00 h. A best-fit model for sand fly density analysis was developed using multiple logistic regression analysis. Geographical information system based spatial analysis techniques were employed to map the household type distribution of insecticide susceptibility of the vector, and IRS-status of the households to interpret the spatio-temporal distributions of *P. argentipes*.

**Results:**

*Phlebotomus argentipes* was highly susceptible to SP (100%) but showed high resistance to DDT with a 49.1% mortality rate. SP-IRS has been reported as having better community acceptance than DDT-IRS in all household types. Residual efficacies were varied between wall-surfaces; both insecticides failed to achieve the duration of IRS effectiveness recommended by the WHO. Reduction in *P. argentipes* counts due to SP-IRS was higher than DDT-IRS between household groups (i.e. sprayed and sentinel), in all intervals post-IRS. Combined spatial risk-maps revealed a better control effect of SP-IRS on sand flies than DDT-IRS in all household types risk-zones. The multilevel logistic regression analysis explored five risk-factors that were strongly associated with the density of *P. argentipes*.

**Conclusions:**

The results contribute to furthering current understanding of IRS-practices for control of visceral leishmaniasis in endemic Bihar, which may help in future actions for improvements.

## Background

Visceral leishmaniasis (VL), also known as kala-azar, is an endemic neglected tropical vector-borne disease caused by a protozoan parasite of the genus *Leishmania.* In the Indian subcontinent (ISC), humans are the only reservoir, and the parasite (i.e. *Leishmania donovani*) is transmitted to humans by the bite of an infected female sand fly, *Phlebotomus argentipes* [1, 2]. In India, VL is mainly endemic in the four middle-eastern states: Bihar, Jharkhand, West Bengal and Uttar Pradesh. A few sporadic cases have also been reported from Madhya Pradesh (central-India), Gujarat (western India), Tamil Nadu and Kerala (southern India), and the sub-Himalayan parts of northern India including Himachal Pradesh, Jammu and Kashmir [3]. Among the endemic states, Bihar is highly-endemic with 33 VL affected districts contributing more than 70% of the total Indian cases annually [4]. Approximately 99 million people are at risk in this region, and the average annual incidence is 6752 cases (between 2013 and 2017).

In Bihar and elsewhere in India, control actions against VL rely on the three main strategies: early case detection, effective treatment and vector control using indoor residual spraying (IRS) of insecticides in houses and animal shelters [4, 5]. IRS using dichlorodiphenyltrichloroethane (DDT 50% WP at 1 g ai/m^2^) was successful in VL control in the 1960s as a side effect of antimalarial campaigns and in a programme mode in 1977 and 1992 [5, 6]. However, recent studies confirm the development of widespread resistance in *P. argentipes* against DDT [4, 7, 8]. In 2015, the national vector-borne disease control programme (NVBDCP, New Delhi) has switched over the IRS from DDT to synthetic pyrethroids (SP; alpha-cypermethrin 5% WP at 25 mg ai/m^2^) [7, 9]. The World Health Organization (WHO) has set a VL elimination goal (i.e. < 1 case/10,000 people per year at sub-district/block level) by 2020 [10]. Several studies have reported that IRS is more efficacious than the other vector control methods, causing the highest reduction of sand fly density [11–13]. A recent model has also predicted that an effective IRS by 80% coverage of total household is capable of achieving the elimination target one to three years earlier in a highly endemic condition (i.e. pre-control endemicity level of 5/10,000) [14]. VL affects the poorest of the poor rural communities within the endemic areas, and its vector control relies only on the IRS, but the residual impact of this control measure on different household types has never been investigated under field conditions within the intervention area [15, 16]. Moreover, after intensive VL-control efforts, in a few villages endemicity persists for several years so that they act as hotspot regions [17]. It is therefore imperative to evaluate the residual effect of IRS on different household types for monitoring sand fly density. Furthermore, developing a geospatial risk map at a micro-scale level will help to better understand and monitor sand fly abundance, even after an intervention. A geographical information system (GIS) is a combination of digital cartographical techniques that allows the storage, overlay, manipulation, analysis, retrieval and visualization of various geo-environmental and socio-demographic datasets for multiple purposes [18–20]. A global position system (GPS) is used to examine the spatial location of the earth’s surface components [21, 22]. GIS- and GPS-based spatial modeling tools and techniques have been used in several epidemiological aspects such as spatio-temporal estimation and outbreak prediction of the diseases, implementation and evaluation of control strategies, interaction of the disease-causing organisms and environmental factors, and spatial-risk mapping [20, 23–26]. Information gathered and generated through the geospatial risk maps could potentially enhance prompt and effective control measures.

This study evaluates the residual efficacies and the intervention effects of DDT- and SP-IRS at a household level under the national VL vector control programme in Bihar, India. Additional aims were to develop a combined spatial-risk map and a sand fly density analytical model based on housing characteristics, insecticide susceptibility of the vector and IRS-status of the households to explore the spatio-temporal distributions of sand flies at a micro-scale level.

## Methods

### Study area

**Fig. 1:**
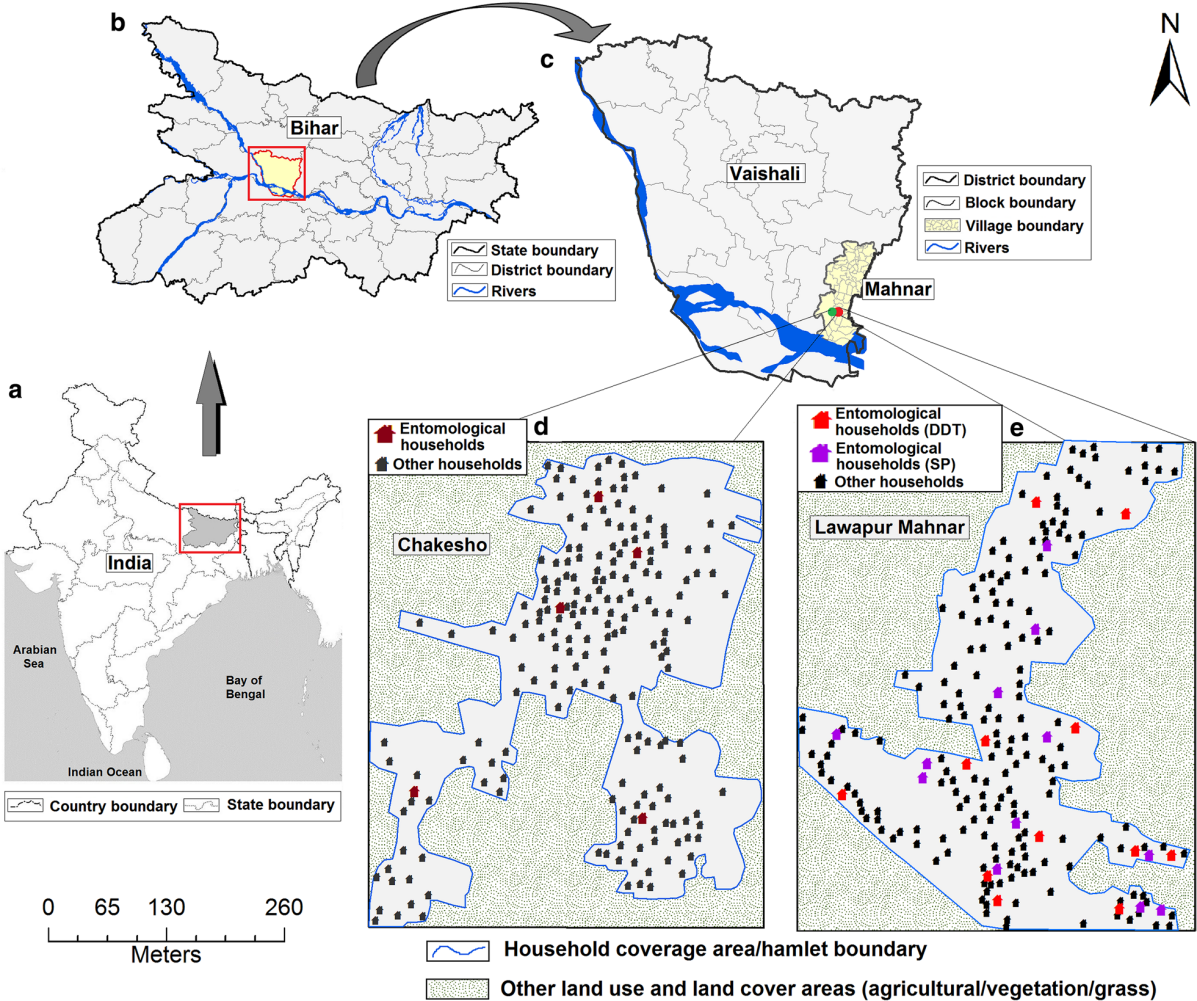
Map of the study area showing the location of Bihar in the India map (**a**) and the location of Vaishali district in the Bihar map (**b**). Two study villages selected in Mahnar block (**c**), i.e. Chakeso as the control site and Lawapur Mahnar as the intervention site

The study was conducted in Mahnar block in Vaishali district on the northern bank of the River Ganges (Fig 1). Mahnar is a highly-endemic zone with an average of 56.7 VL cases per year (170 cases in 2012–2014); annual case incidences range between 2.5 to 3.7 per 10,000 population. Two villages were selected: Chakeso as the control site (Fig. 1d1; no VL case in the past five years) and Lawapur Mahanar as the endemic site (Fig. 1d2; highly endemic, 5 or more than 5 cases per 1000 population per year in the past5 years). Village selection was based on three main criteria: geographical location and easy accessibility (i.e. riverside and easy accessibility throughout the year), population characteristics and household numbers (i.e. minimum 200 households; 202 and 204 households with average of 4.9 and 5.1 family sizes for Chakeso and Lawapur Mahanar, respectively) and household types (HTs) and its distribution pattern (i.e. mixed HTs with random distribution). Both the study villages are situated within 500 m of the Mahnar town and the block hospital. Villagers of the study villages were found to be very cooperative with the study activities. Houses [consisting of 1–2 bedrooms attached with one veranda, one kitchen, one bathroom and one cattle shed (attached or separated)] in the study villages are made of brick/mud walls with mudplaster and a mudfloor, brickwalls with lime-coated cement plaster and a cement floor, unplastered and unpainted brick-walls and a mud floor, and thatched. The entire Vaishali district is characterized by a humid sub-tropical climate, with one rainy season (July–August) and one dry season (November–December). The average annual rainfall is 720.4 mm (range: 736.5–1076.7 mm) with a relative humidity of 65 ± 5% (range: 16–79%) and an average monthly temperature ranging between 17.2–32.4 °C. May and June (temperature ranging between 39–44 °C) are the warmest months and January is the coldest month (temperature ranging between 7–22 °C).

### IRS implementation and monitoring

As a part of the national kala-azar control programme, two rounds (first round, February–March; second round, June–July) of annual IRS during 2015 and 2016 were conducted by the State Health Society of Bihar (SHSB) [4]. To ensure effective implementation of all IRS-activities a micro-action plan was prepared by Rajendra Memorial Research Institute of Medical Sciences, Patna (RMRIMS; Bihar), a regional nodal institute of Indian Council of Medical Research (ICMR; New Delhi). IRS villages were selected based on two main criteria: a past history of VL and post-dermal kala-azar (PKDL) cases in the village (i.e. villages having one or more cases in any period of time in the last 3 years, including the implementing year), and the peripheral non-endemic villages of the ‘hotspot’ (i.e. villages either continued case report for ≥ 2 years or reported ≥ 2 cases per 1000 population) and the newly-endemic (no case in the past 3 years of the study year) villages reported in the last year of the implementation year [17]. Neighboring villages of the newly endemic villages during the first round of IRS in the implementing year were also included in the action plan of second round IRS. In 2015, in the intervention study village, both the IRS rounds were performed by DDT (DDT 50% WP at 1 g ai/m^2^). From 2016 onwards, IRS was conducted using a synthetic pyrethroid (SP; alpha-cypermethrin 5% WP at 25 mg ai/m^2^). Hudson Xpert pumps (13.4 l) with pressure gauze, a control flow valve (at 1.5 bar) and a flat fan 8002 nozzles for porous surfaces were used for spraying [27]. ICMR-RMRIMS, Patna (Bihar) conducted the IRS monitoring activities at the household and village levels, and also provided prior information of IRS to the villagers through mike 1–2 day before. A monitor for each IRS-squad was employed (by RMRIMS) for monitoring the IRS squad work. Monitors were moved with the IRS squads in all households for providing beneficial effects of IRS to household-heads and convinced them accordingly. A minimum 80% coverage of total households was confirmed in the study village during both IRS rounds [4]. Spraying status (i.e. refused, partially- and fully-sprayed; defined in Additional file 1: Table S1) of all households in intervention study village was recorded for both IRS-rounds.

### Baseline data collection and post-IRS household selection for entomological survey

This study was conducted between June 2015 and July 2016. IRS pre (i.e. 2 weeks before intervention; baseline survey) and post (i.e. 2, 4 and 12 weeks after intervention; follow-up surveys) sand fly densities were monitored using Centers for Disease Control and Prevention light traps one night (i.e. from 18:00 to 6:00 h) for each household in each IRS-round [28]. Light traps were installed in bedrooms and animal shelters. In the intervention study village, pre-IRS sand fly densities were monitored in 48 households (12 households/day for 4-days continuously until the day before IRS-day). Twelve households each from the four main types household groups [i.e. plain mud plastered (PMP), cement plastered and lime-coated (CPLC), brick unplastered and unpainted (BUU), and thatched (TH)] were selected. Thereafter, only 12 households (from the 48 pre-IRS households) were selected for continuing sand fly density collection in post-IRS session. Six households each from the intervention (households treated by IRS) and the sentinel (household in intervention village, those owners refused permission for IRS) groups were selected based on the WHO-guidelines [28]. In the control group (households in an adjacent village where IRS is not performed due to the absence of VL), only 6 households were selected for sand fly density monitoring before and after both IRS sessions. For all three sand fly density monitoring groups (i.e. intervention, sentinel and control), households were selected from the three risk-levels groups (i.e. low, medium and high; two households from each risk level) were categorized based on the HTs risk characteristics (module and structured in Tables 1 and 2, respectively) [29, 30]. Two households from each risk-level were selected to avoid bias in sand fly density estimation for comparing the results between groups. In the intervention group, post-IRS sand fly densities were monitored in two types of IRS households: fully sprayed (*n *= 3; 1 household from each level of risk group) and partially sprayed (*n *= 3; 1 household from each level of risk group).

**Table 1:** Different types of household level risk factors associated with *P. argentipes* abundance identified for risk scoring

Household level risk factor	Subcategory	Risk score
A. Type of floor (TF)^a,b^	A1. Cemented	0
	A2. Mud	1
B. Type of wall (TW)^a,b^	B1. Brick with cement plastered	0
	B2. Brick	1
	B3. Thatched	2
	B4. Mud and brick with mud plastered	3
C. Type of roof (TR)^a,b^	C1. Cemented and asbestos	0
	C2. Tiles and cuprile	1
	C3. Thatched	2
D. Dwelling status (DS)^a,b^	D1. No cattle shed	0
	D2. Separate dwelling	1
	D3. Mixed dwelling/attached cattle shed	2
E. IRS-status (IRSS)^c^	E1. Not sprayed	2
	E2. Partially sprayed	1
	E3. Fully sprayed	0
F. Insecticide susceptibility of vector (ISV)	F1. < 90% mortality	2
	F2. 90–98% mortality	1
	F3.> 98% mortality	0

^a^HT-risk scores for the groups ‘A–D’ were based on household suitability for sand flies breeding and resting characteristics [26, 29, 30]

^b^Groups ‘A–D’ for both invention and control sites for entomological household selection

^c^Group ‘E’ for intervention site for entomological household selection

**Table 2:** Household type-, IRS-status- and insecticide susceptibility-based risk combinations (at high, medium and low levels) developed for risk-index calculation

Level of risk	Score range	Risk combinations
HT-based risk index		
Low	1–2	(A1 + B1 + C1 + D1); (A2 + B1 + C1 + D1); (A2 + B2 + C1 + D1); (A1 + B2 + C2 + D1); (A1 + B1 + C2 + D2); (A2 + B1 + C2 + D1); (A2 + B1 + C1 + D2); (A1 + B2 + C1 + D2); (A1 + B1 + C1 + D3)
Medium	3–4	(A2 + B2 + C2 + D2); (A2 + B2 + C2 + D1); (A2 + B2 + C1 + D2); (A2 + B1 + C2 + D2); (A1 + B2 + C2 + D2); (A2 + B1 + C1 + D3); (A1 + B2 + C1 + D3); (A1 + B1 + C2 + D3); (A2 + B3 + C2 + D1)
High	5–8	(A2 + B2 + C2 + D3); (A2 + B3 + C3 + D1); (A2 + B3 + C3 + D2); (A2 + B3 + C3 + D3); (A2 + B3 + C2 + D3); (A2 + B4 + C2 + D1); (A2 + B4 + C2 + D2); (A2 + B4 + C2 + D3); (A2 + B4 + C3 + D1); (A2 + B4 + C3 + D2); (A2 + B4 + C3 + D3)
IRS status (IRSS)-based risk index		
No risk	0	E3
Medium	1	E2
High	2	E1
Vector susceptibility to insecticides (VSI)-based risk index		
Mortality> 98%	0	F3
Mortality 90–98%	1	F2
Mortality < 90%	2	F1

### Sand fly identification; population density and reduction calculations

All field caught sand flies collected in a test tube was transferred to the laboratory and killed using chloroform-soaked cotton-wool to the test tubes. *Phlebotomus argentipes* was identified and segregated by sex, from other insects and sand flies based on morphological characters using standard identification keys [31]. All male and female *P. argentipes* were then preserved separately in 80% alcohol. Sand fly density was calculated at per-trap/per-night using the formula as follows: the total number of sand flies collected/the number of light traps installed for a single night. The percent change of sand fly count (SFC) due to IRS using DDT and SP was estimated using the following formula [32]:

$$\% {\text{ of change }}\left( {\text{R}} \right) = \left[ {\left( {{\text{B}} - {\text{A}}} \right) - \left( {{\text{D}} - {\text{C}}} \right)/{\text{A}}} \right] \times 100$$

where A is the baseline mean SFC for the intervention household, B is the post-IRS mean SFC for the intervention household, C is the baseline mean SFC for the control/sentinel household, and D is the post-IRS mean SFC for the control/sentinel household.

The result of intervention effect recorded with negative and positive values indicates a decrease and increase in SFC after IRS, respectively. Intervention effect calculated zero if the post-IRS SFC remains same with baseline SFC.

### Sand fly susceptibility test to insecticides

The susceptibility of local *P. argentipes* to the insecticides DDT and SP was assessed using the standard tube-bioassay method based on WHO pesticide evaluation scheme (WHOPES) [33]. Healthy and unfed females of *P. argentipes* (18–25 SFs for each set) were exposed to insecticide-impregnated papers of DDT(4%) and SP (alpha-cypermethrin 0.05%) procured from the University Sains Malaysia (USM, Malaysia; coordinated by the WHO) using a WHO insecticide susceptibility evaluation test kit [4, 9, 33, 34]. Eight tests (four test replicates and one control for each test, simultaneously) were performed for each set of insecticide bioassay. Control tests were performed using risella (for DDT) and silicone oil (for SP) pre-impregnated papers supplied by USM. After 60 min of exposure, sand flies were transferred to WHO-holding tubes and provided with cotton wool soaked with 10% sugar solution. The number of sand flies “knocked down” at 1 h and final mortality rates at 24 h were observed. The resistance status was described according to WHO guidelines: 98–100% mortality indicates susceptibility, 90–98% indicates the possibility of resistance that needs to be confirmed and < 90% indicates resistance [33, 34]. No mortality correction was performed as the control mortality rates ranged between 0–5%.

### Bioavailability of insecticide

The bioefficacy and the residual effect of the insecticides against the local *P. argentipes* under field conditions were assessed. Standard WHO cone bioassays were performed at 2, 4 and 12 weeks after spraying in the three intervention households (one household each from plain mud-plastered or PMP, cement plastered and lime coated or CPLC, brick unplastered and unpainted or BUU) in which light traps were installed [27, 32]. TH households were omitted for their uneven wall surface. In each assay, 12 cones (four cones per household and one household for each type of wall-surface) were used for all experimental households. Cones were fixed on each wall in a room at different heights: one at head height (between 1.7 and 1.8 m), two at waist height (between 0.9 and 1 m) and one below knee height (between 0.3 and 0.5 m. Ten unfed female sand flies (10 sand flies for each cone; collected by aspirator from the control site) were introduced in each WHO-plastic cone. Three-cone chambers (one cone for each type of household) were used as control; sand flies were exposed to the unsprayed walls. After 30 min of exposure, sand flies were carefully pulled out from the cone chambers with the help of a bent-end aspirator and transferred to WHO-holding tubes equipped with 10% sugar solution for nourishment. All tubes were stored in a place maintained at a temperature of 27 ± 2 °C and 80 ± 10% relative humidity to record the final mortality after 24 h. The mortality rates scored between 5% and 20% was corrected by Abbott’s formula [27] as follows:

$$P = \frac{P1 - C}{100 - C} \times 100$$

where P is the corrected mortality, P_1_ is the percent observed mortality and C is the percent mortality in the control. Test results scored with control mortality > 20% were cancelled and re-performed [27, 33].

### Geo-database generation, layer integration and spatial risk-map preparation

A comprehensive household survey was conducted in the intervention study village. The GPS location of each household along with its construction and material types, dwelling and intervention status were recorded. A digital geo-database including village, block, district and state levels boundary layers was developed in the GIS-platform. All household locations were geo-tagged using a point-GIS layer at the village level, and their attribute information was linked and updated. Each household point was given a risk score based on HTs, insecticide susceptibility of vector and IRS-status (Table 1) [11, 26, 29, 30]. All household location points were then converted into a thematic map using inverse distance weighting (IDW; resolution of 6 m^2^ based on average household area, power of 2, number of surrounding points fixed= 10, using a variable search radius, low-pass filter, and cubic convolution display) spatial interpolation technique [35]. Two types of thematic spatial risk-maps were generated: a HT-based thematic map and an insecticide susceptibility of vector and IRS-status (ISV & IRSS)-based thematic map. Both the thematic risk-maps were then combined using weighted overlay analysis [36]. In this process, raster layers were reclassified into a common preference scale of different risk levels (i.e. high, medium and low/no-risk). Each reclassified raster layer was then multiplied by its assigned weight, taking into consideration of relative importance of parameters which furnishes favorable conditions (based on the endemic status of the study village, sand fly breeding sites, and resting and feeding behavior) for *P. argentipes* abundance [26, 29, 30, 37]. A 50:50 weight to both thematic risk-maps was given as they equally contributed to sand fly abundance (Additional file 1: Table S2). By adding-up both the weighted overlay thematic maps, a final combined-risk map was generated and visualized at a GIS platform. The final risk-map was presented and described with a sand fly risk index (SFRI) value, estimated using the following equation:

$$P = \frac{\varSigma L}{H} \times 100$$

where P is the risk index value, L is the total risk value of the respective household location and H is the highest household risk value in the study area. We used ESRI ArcGIS v.9.3 (Redlands, CA, USA) to prepare and perform the GIS layers and analyses, in order to produce the risk maps.

### Household characteristics and IRS-intervention-based sand fly density analysis

A multiple regression analysis was performed to explore the combined effect of HTs and ISV & IRSS (described in Table 1) on sand fly densities in the households (*n *= 24). The housing characteristics and the IRS-intervention-based risk factors recorded in the study were considered as explanatory variables, while sand fly density was used as response variables. Poisson regression-based univariate analysis was performed for each explanatory variable with the sand fly density. Variables, which were non-significant and recorded with *P*-values higher than 15% during the univariate analysis, were removed for the multiple regression analysis. To check the interaction effect, the interaction terms of all the possible combination of significant variables (found in univariate analysis) were included simultaneously in the multiple regression analysis, and non-significant terms were removed stepwise from the model in order to produce the final model.

### Evaluation of household risk-scores and risk-map zones, and validation of the sand fly density analytical model

The household-based sand fly-risk assessment was performed in two ways: evaluation of household level risk score and evaluation of combined map spatial risk-zones. The household level risk score was evaluated using correlation analysis between household risk scores and sand fly densities (collected from 6 sentinel and 6 intervention households; in pre- and post-IRS weeks). Spatial risk-zones, evaluated using the mean number of sand flies collected in different households, were compared between risk groups (i.e. low, medium and high-level zones). In each IRS-round, 12 households (4 households each from the three levels of risk zones; single night collection at 2-, 4- and 12-week intervals post-IRS) were randomly selected for sand fly collection to validate the combined risk-map. The same household data (i.e. HTs, VSI, IRSS and mean sand fly densities) were used for validation of the final regression model. Simple correlation analysis was performed between field observed, and model predicted sand fly densities in the households.

### Statistical analysis

Descriptive statistics such as mean, minimum, maximum, 95% confidence interval (CI) and percentage were calculated to summarize entomological and IRS-related data. The mean *P. argentipes* count/density between IRS-household groups (i.e. sprayed *vs* sentinel, sprayed *vs* control, sentinel *vs* control, and fully *vs* partially), between IRS-rounds (i.e. DDT *vs* SP) and mortality rates (for insecticide residual effectiveness) between household surface types (i.e. BUU *vs* CPLC, BUU *vs* PMP and CPLC *vs* PMP) were compared using a parametric test [paired samples t-test (for normally distributed data)] and a non-parametric test (Wilcoxon signed-rank test for non-normally distributed data). All analyses were carried out by using SPSS software v.20 (SPSS Inc., Chicago, IL, USA).

## Results

### Household coverage estimation during IRS

Household coverage in the intervention study village during DDT and SP IRS-rounds were calculated. A total of 205 households were targeted for IRS in each round, of which 179 households (87.3%) in the DDT-round and 194 households (94.6%) in the SP-round accepted IRS for VL vector control. The percentage of fully sprayed households during SP-IRS (86.3%) was higher than the DDT-IRS (52.7%). The number of households which refused IRS during both the IRS-rounds were 26 (12.7%) during DDT and 11 (5.4%) during SP. The number of partially sprayed households recorded were 71 (34.6% of the total sprayed households) and 17 (8.3% of the total sprayed households) during DDT and SP rounds, respectively.

### *Phlebotomus argentipes* susceptibility to DDT and alpha-cypermethrin

Based on the WHO insecticide resistance guidelines, the *P. argentipes* population was fully susceptible to alpha-cypermethrin (0.05%) in the intervention site as the average test mortality recorded (at 24 h) was 100%. The observed knockdown rate was 85.9% (95% CI: 81.1–90.6%). For DDT, the knockdown rate was 22.8% (95% CI: 11.5–34.1%) and the average e test mortality was 49.1% (95% CI: 41.9–56.3%) after 24 h. Results showed that *P. argentipes* from the intervention site had developed complete resistance to DDT.

### Insecticide residual effectiveness

Results of cone bioassays on different surface types (at different time interval post–IRS) sprayed with DDT and SP are summarized in Table 3. Our data showed that after 24 h mortality rates varied between wall-surface types for both the insecticides (BUU *vs* CPLC: *t*_(2)_= − 6.42, *P *= 0.02; BUU *vs* PMP: *t*_(2)_= 0.25, *P *= 0.83; CPLC *vs* PMP: *t*_(2)_= 1.03, *P *= 0.41 for DDT-IRS and BUU *vs* CPLC: *t*_(2)_= − 5.86, *P *= 0.03; BUU *vs* PMP: *t*_(2)_= 1.42, *P *= 0.29; CPLC *vs* PMP: *t*_(2)_= 3.01, *P *= 0.10 for SP-IRS; overall DDT *vs* SP: *t*_(2)_= 9.70, *P *= 0.01). Mortality rates decreased steadily with time intervals. For SP-IRS, a satisfactory percentage (i.e. ≥ 80% as per WHO) of mortality was recorded for all wall-surface types, 2 weeks after spraying (i.e. overall 95.6%) and 4 weeks after spraying for CPLC walls only (i.e. 82.5%). For DDT groups, mortality rates were always recorded below 70% for all wall-surface types during all time intervals post-IRS bioassays. The average test mortality for DDT and SP 12 weeks after spraying were 25.1% and 63.2%, respectively. The highest mean mortality for DDT, on all three surface-types, were 61.1% (for PMP, at 2 weeks post-IRS), 36.9% (for CPLC, at 4 weeks post-IRS) and 28.9% (for CPLC, at 12 weeks post-IRS); the lowest rates were 55% (for BUU, at 2 weeks post-IRS), 32.5% (for PMP, at 4 weeks post-IRS) and 20% (for PMP, at 12 weeks post-IRS). For SP, the highest mean mortality rates for all surface-types were 97.2% (for CPLC, at 2 weeks post-IRS), 82.5% (for CPLC, at 4 weeks post-IRS) and 67.5% (for CPLC, at 12 weeks post-IRS); the lowest rates were 94.4% (for BUU, at 2 weeks post-IRS), 75% (for PMP, at 4 weeks post-IRS) and 58.3% (for PMP, at 12 weeks post-IRS).The fall-off in the mortality rate with time intervals for PMP sprayed surfaces was faster than the CPLC and BUU sprayed surfaces, for both the insecticides.

**Table 3:** The mortality rate (in %) of *P. argentipes* evaluated through cone bioassays on different wall surface types at 2, 4 and 12 weeks DDT- and SP-post-IRS in Lawapur Mahnar village, Vaishali district (Bihar)

Insecticide sprayed	Surface/data type	Time interval post-IRS		
		2 weeks Mean (95% CI)	4 weeks Mean (95% CI)	12 weeks Mean (95% CI)
DDT (WP 50%) at 1 g/m	Brick unplastered and unpainted (BUU)	55 (50.0–60.0)	35 (30.0–40.0)	26.4 (22.2–33.3)
	Cement plastered and lime-coated (CPLC)	58.3 (55.6–66.7)	36.9 (30.0–44.4)	28.9 (22.2–30.3)
	Plain mud plastered (PMP)	66.1 (55.6–66.7)	32.5 (20.0–40.0)	20 (10.0–30.0)
	Overall (average test mortality)	58.1 (50.0–66.7)	34.8 (20.0–40.4)	25.1 (10.0–33.3)
SP (WP 5%) at 25 mg/m	Brick unplastered and unpainted (BUU)	94.4 (88.9–100)	77.5 (70.0–80.0)	63.9 (44.4–77.8)
	Cement plastered and lime-coated (CPLC)	97.2 (88.9–100)	82.5 (70.0–90.0)	67.5 (60.0–80.0)
	Plain mud plastered (PMP)	95 (90.0–100)	75 (66.7–88.9)	58.3 (44.4–66.7)
	Overall (average test mortality)	95.6 (88.9–100)	78.3 (66.7–90.0)	63.2 (44.4–80.0)

### IRS intervention effect

The intervention effect (i.e. change in sand fly abundance after IRS) of DDT- and SP-based IRS-rounds are summarized in the Table 4 (Additional file 1: Figure S1). For DDT-IRS, the percent reduction in *P. argentipes* counts at post-IRS intervals were 34.1% (after 2 weeks), 25.9% (after 4 weeks) and 14.1% (after 12 weeks). For SP-IRS, reduction rates were 90.5% (after 2 weeks), 66.7% (after 4 weeks) and 55.6% (after 12 weeks). The highest reduction in *P. argentipes* count in sentinel households during DDT- and SP post-IRS recorded were 2.8% (after 2 weeks) and 49.1% (after 2 weeks), respectively. Reductions in *P. argentipes* count (pre *vs* post) in both sprayed (*t*_(2)_= − 9.09, *P* < 0.001) and sentinel (*t*_(2)_= − 1.29, *P *= 0.33) households were recorded higher during SP-IRS compared to DDT-IRS, in all 3 time intervals post–IRS. For both insecticides, the *P. argentipes* count was increased (i.e. 3.6% and 9.9% for SP and DDT, respectively) in the sentinel households at 12 weeks post-IRS. A total of 112 and 161 *P. argentipes* were collected from the sentinel households during SP and DDT post-IRS session, respectively.

**Table 4:** The number of *P. argentipes* collected in sprayed, sentinel and control households in pre- and post-IRS weeks during DDT-IRS and SP-IRS rounds. Percentage reduction calculated in sprayed and sentinel households compared with control households

Type	Weeks	*P. argentipes* count at intervention and control sites			Post-intervention reduction	
		Sprayed households Mean (95% CI)	Sentinel households Mean (95% CI)	Control households Mean (95% CI)	Sprayed households (%)	Sentinel households (%)
DDT (WP 50%) at 1 g/m^2^						
Pre-IRS DDT	2	14.2 (1–28)	11.8 (2–25)	12.3 (2–14)	–	–
Date of IRS	25.06.15					
Post-IRS DDT	2	6.2 (5–14)	8.3 (1–17)	9.2 (8–20)	− 34.1	− 2.8
	4	6.5 (1–14)	7.7 (1–15)	8.3 (1–18)	− 25.9	− 1.4
	12	9.5 (1–16)	10.3 (2–22)	9.7 (9–20)	− 14.1	9.9
SP (WP 5%) at 25 mg/m^2^						
Pre-IRS SP	2	10.5 (1–21)	9.2 (1–22)	7.8 (1–17)	–	–
Date of IRS	22.04.16					
Post-IRS SP	2	0.0 (0–0)	3.7 (1–8)	6.8 (3–12)	− 90.5	− 49.1
	4	2.3 (2–5)	6.2 (5–12)	6.7 (5–13)	− 66.7	− 20.0
	12	8.5 (3–15)	13.3 (2–27)	11.7 (1–22)	− 55.6	3.6

### Sand fly abundance between household groups in pre- and post-IRS weeks

**Fig. 2:**
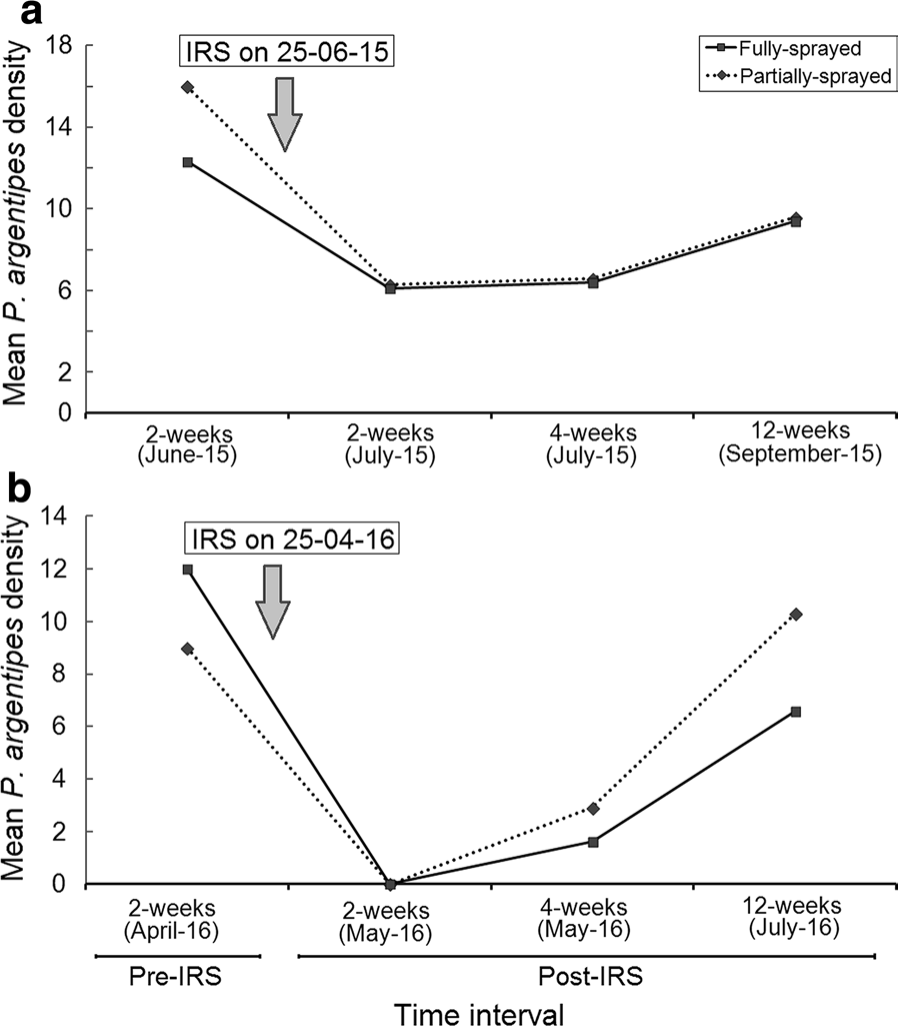
Mean *P. argentipes* densities calculated in fully- and partially sprayed households in the Lawapur Mahanar village at 2 weeks pre-IRS and 2, 4 and 12 weeks post-IRS during DDT- and SP-rounds

No significant difference in *P. argentipes* density was observed between household groups (i.e. sprayed *vs* sentinel: *t*_(2)_= − 3.47, *P *= 0.07; sprayed *vs* control: *t*_(2)_= − 2.03, *P *= 0.18; sentinel *vs* control: *t*_(2)_= − 0.59, *P *= 0.62) during DDT post-IRS weeks. Conversely, a significant difference in *P. argentipes* density was observed between the sprayed *vs* sentinel (*t*_(2)_= − 11.28, *P *= 0.01) and sprayed *vs* control (*t*_(2)_= − 4.42, *P *= 0.05) households groups during SP post-IRS weeks. No significant difference was observed between sentinel and control households (*t*_(2)_= − 0.48, *P *= 0.68) for SP-IRS. Mean *P. argentipes* densities observed in fully- and partially sprayed households for both the IRS-rounds are presented in Fig. 2. No significant difference in *P. argentipes* density was observed between fully- (mean 7.3 and 2.7 per-trap/night for DDT-IRS and SP-IRS, respectively) and partially-sprayed (mean 7.5 and 4.4 per-trap/night for DDT-IRS and SP-IRS, respectively) households in post-IRS weeks for both the insecticides (*t*_(2)_ *≤* 1.0, *P *> 0.2). However, the *P. argentipes* densities in fully- and partially-sprayed households varied significantly between SP and DDT IRS-rounds (*t*_(2)_ ≥ 4.54, *P* ≤ 0.05).

### Risk-zone mapping, visualization and sand fly density monitoring

**Fig. 3:**
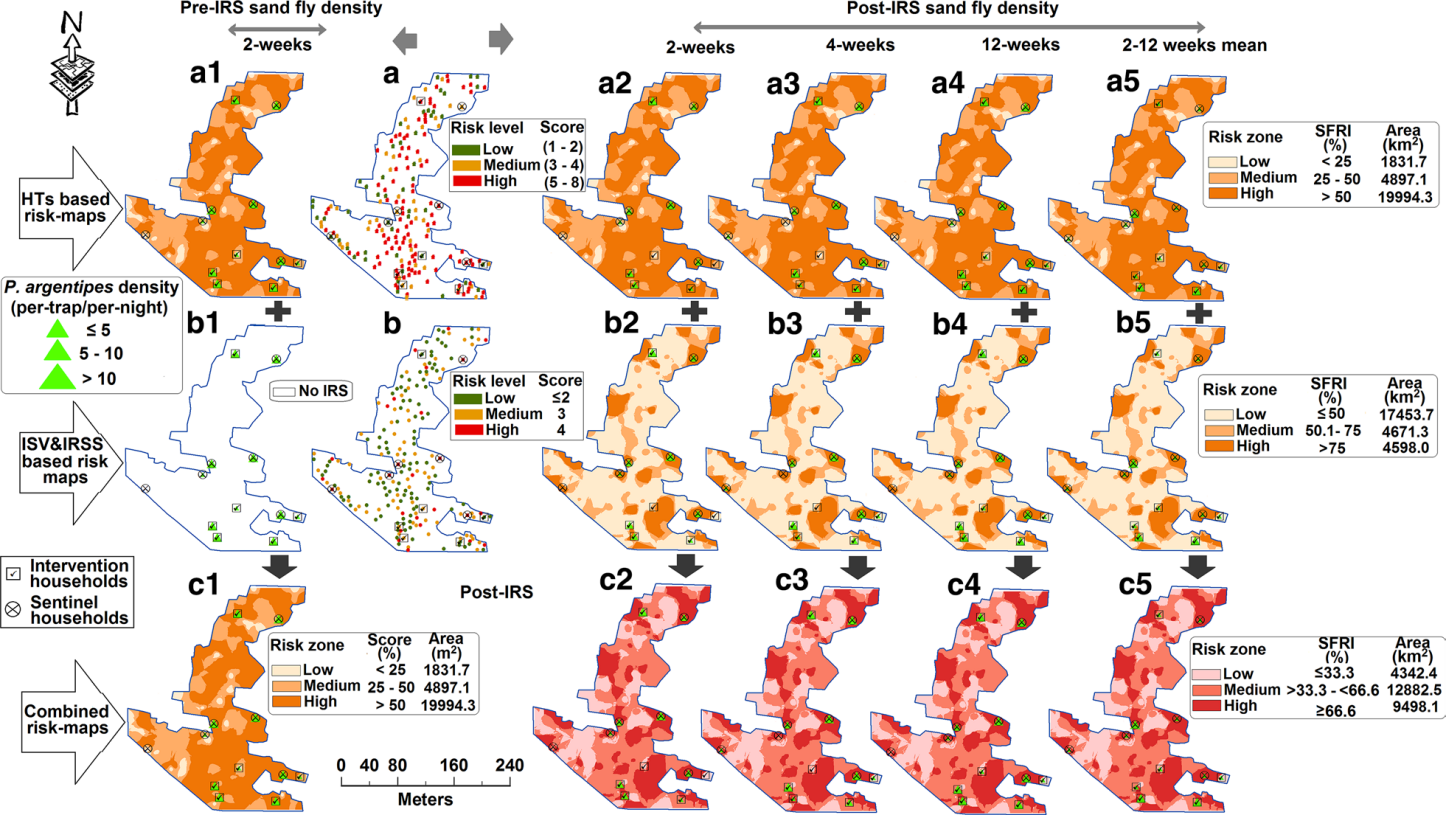
GIS-based three types of spatial risk maps (i.e. HTs, IS & IRSSs and a combination of HTs and IS & IRSSs) developed for *P. argentipes* risk-zone identification pre- and post-DDT-IRS in Lawapur Mahnar village, Vaishali district (Bihar)

**Fig. 4:**
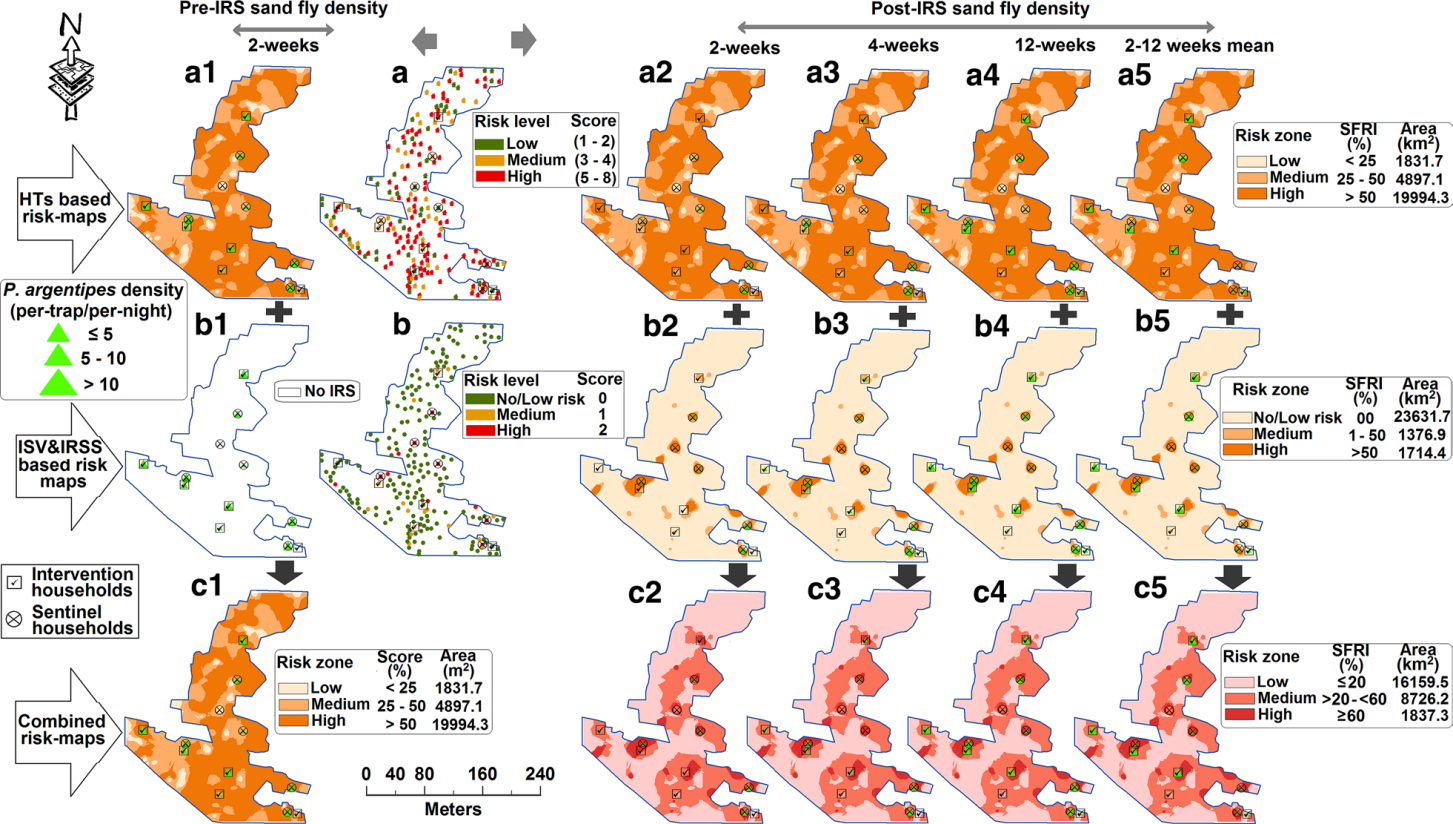
GIS-based three types of spatial-risk maps (i.e. HTs, IS & IRSSs and a combination of HTs and IS & IRSSs) developed for *P. argentipes* risk-zone identification pre- and post-SP-IRS in Lawapur Mahnar village, Vaishali district (Bihar)

**Fig. 5:**
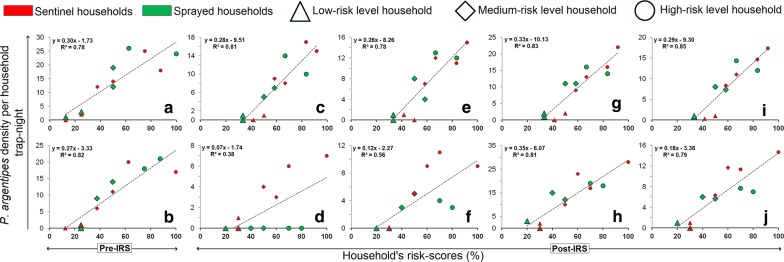
Effect of DDT- (**a**, **c**, **e**, **g**, **i**) and SP-IRS (**b**, **d**, **f**, **h**, **j**) on different levels of household-type risk groups evaluated through ‘*R*^2^’ calculation between the estimated household risk score and the mean *P. argentipes* density of the households at 2 weeks pre-IRS and at 2, 4 and 12 weeks post-IRS in Lawapur Mahnar village, Vaishali district (Bihar)

A combined spatial risk-map (for Lawapur Mahanar village; total area: 26,723 km^2^) was developed for identifying low, medium and high levels of spatial risk-zones for monitoring *P. Argentipes* emergence and resurgence in pre- and post-IRS weeks (Figs. 3, 4). The highest risk-score estimated for a household for the spatial risk map generation was ‘12’ (i.e. ‘8’ for HTs-based risk map and ‘4’ for VSI & IRSS-based risk map). The lowest risk score calculated was ‘zero’ or ‘no risk’ except for the DDT-VSI & IRSS map which had a lowest score of 1. The HT-based risk map revealed that a large extent (i.e. 19,994.3 km^2^; 74.8%) of the Lawapur Mahanar village was under the high-risk zone and the households of this zone had the maximum chance of emergence and resurgence of sand flies. Area coverage of high (20.2% for DDT; 4.9% for SP), medium (22.3% for DDT; 4.6% for SP) and low/no-risk (57.5% for DDT; 90.5% for SP) zones varied (*t*_(2)_= 12.7, *P* < 0.05) between DDT- and SP-IS & IRSS risk-maps (Figs. 3, 4). Developed final combined risk-maps showed that the SP-IRS had better protection capability than the DDT-IRS in all levels of HT-based risk-zones. HTs-based high-risk area had diminished at below the 7% (1837.3 km^2^) after SP-IRS, and most of the area (i.e. 53.6%) was converted into a low risk-zone. During DDT-IRS, percentage of high- and low-risk areas estimated through the combined risk-map were 35.5% (9498.1 km^2^) and 16.2% (4342.4 km^2^), respectively. *Phlebotomus argentipes* densities measured in the sprayed and the sentinel households in pre- and post-IRS weeks were mapped and visualized on the combined risk-maps for both IRS rounds (i.e. DDT and SP) (Figs. 3, 4). A good agreement was observed between the household risk-scores and the mean *P. argentipes* densities recorded in pre- and post-IRS periods (Fig 5). The *R*^2^ values (at *P* < 0.05) of agreement analyses for both the IRS-rounds computed were 0.78 for pre-DDT 2-weeks, 0.81 for post-DDT 2-weeks, 0.78 for post-DDT 4-weeks, 0.83 for post-DDT 12-weeks, 0.85 for post-DDT overall, 0.82 for pre-SP 2-weeks, 0.38 for post-SP 2-weeks, 0.56 for post-SP 4-weeks, 0.81 for post-SP 12-weeks, and 0.79 for post-SP overall (Additional file 1: Table S3). Results revealed an enhanced intervention effect of SP-IRS on all HTs up to 4-weeks post-IRS. The DDT-IRS had remained ineffective on all HTs at all intervals post-IRS. Field evaluation results of combined risk-map zones are summarized in Table 5. For both the IRS-rounds, mean counts of *P. argentipes* and its percentage (of the total count) in the high-risk zone (i.e.> 55%) were higher than the low and the medium levels risk-zones, for all time intervals post-IRS. Locations of entomological households (i.e. the households selected for sand fly collection) are mapped and visualized in Additional file 1: Figure S2.

**Table 5:** The number of *P. argentipes* collected in low, medium and high risk-levels households after DDT-IRS and SP-IRS rounds in Lawapur Mahnar village, Vaishali district (Bihar)

Risk group	2 weeks Mean (95% CI)	4 weeks Mean (95% CI)	12 weeks Mean (95% CI)	Overall Mean (95% CI)	Total count (%)
DDT					
Low	0.8 (0–2)	1.3 (0–4)	1.0 (1–3)	1.0 (0–4)	12 (4.4)
Medium	4.5 (1–7)	8.8 (6–14)	13.5 (6–23)	8.9 (1–23)	107 (39.5)
High	7.3 (2–12)	11.3 (6–17)	19.5 (11–27)	12.7 (2–27)	152 (56.1)
SP					
Low	0 (0–0)	0.3 (0–1)	1.0 (0–2)	0.4 (0–2)	5 (3.1)
Medium	0.3 (0–1)	3.3 (1–6)	12.5 (7–16)	5.3 (0–16)	64 (40.3)
High	0.5 (0–2)	4.8 (3–8)	17.3 (10–26)	7.5 (0–26)	90 (56.6)

### *Phlebotomus argentipes* density analysis based on HTs and IRS risk-factors

Results of univariate analysis of all the risk factors to the *P. argentipes* densities are summarized in Table 6. All risk-factors (*n *= 6) were found significantly associated with sand fly densities in the households. The significance level of all associated variables was observed to produce *P*-values less than 0.15. Therefore, all explanatory variables were retained for multiple regression analysis. The best fit combination for the final model was produced with five risk-factors: TF, TW, DS, ISV and IRSS. Details of parameters selected in the final model are presented in Table 7 with adjusted odds ratios with 95% confidence intervals (CI) and *P*-values. The final model was highly significant, with a *R*^2^ value of 0.89 (*F*_(5)_= 27.9, *P* < 0.001), and the model formulated was:

$${\text{Sand fly density}} = 2. 9\times {\text{THF}} + 1. 5\times {\text{TW}} + 2. 1\times {\text{DS}} + 1. 3\times {\text{ISV}} + 1. 7\times {\text{IRSs}} - 1 3.0$$

**Table 6:** Association of household type and IRS risk-factors with *P. argentipes* densities in Lawapur Mahanar village of Mahnar block, Vaishali district (Bihar) evaluated using Poisson regression based univariate analysis

Variables	Crude odds ratio (95% CI)	*P-*value
Type of floor (TF)	5.4 (4.2–6.9)	< 0.001
Type of wall (TW)	4.9 (4.0–6.1)	< 0.001
Type of roof (TR)	5.4 (4.3–6.8)	< 0.001
Dwelling status (DS)	4.4 (3.5–5.6)	< 0.001
Insecticide susceptibility of vector (ISV)	4.8 (3.8–6.0)	< 0.001
IRS-status (IRSS)	6.1 (4.8–7.7)	< 0.001

**Table 7:** Analysis of *P. argentipes* density in response to household type and IRS risk-factors in Lawapur Mahanar village of Mahnar block, Vaishali district (Bihar) using multiple regression model

Variables	Adjusted odds ratio (95% CI)	*P-*value
Type of floor (TF)	2.9 (0.4–5.4)	< 0.001
Type of wall (TW)	1.5 (0.2–2.8)	< 0.001
Dwelling status (DS)	2.1 (0.4–3.8)	< 0.001
Insecticide susceptibility of vector (ISV)	1.3 (0.1–2.6)	0.04
IRS-status (IRSs)	1.7 (0.9–2.5)	< 0.001

TR was deselected in the final model for its least significance (*P *= 0.46) with other explanatory variables. The developed model was used to predict sand fly density using the data of 12 different households. Validation result showed a strong correlation between field observed and model predicted sand fly densities (*r *= 0.91, *P* < 0.001).

## Discussion

The elimination of VL in the endemic Indian states is targeted by 2020 [10]. From 2012 onwards, India has made substantial progress in reduction of VL cases and deaths [10]. In 2015, the switchover from DDT to SP was an important change in the IRS history of Bihar, India [38]. To understand the spatial risk of VL and its vector abundance, several studies have been performed at a macro-scale level. However, little research has been conducted on a micro-scale level, although the spatial distribution of VL endemicity becomes increasingly focal around the country. Moreover, at a micro-scale level, the evidence is less consistent and more challenging to analyze and understand. To our knowledge, the present study is the first report on the evaluation of the residual efficacy and interventional effect of IRS using DDT and SP insecticides between HTs under the national VL vector control programme in Bihar state (India). It is also the first attempt to develop a spatial-risk map and a sand fly density analytical model to reveal spatio-temporal distributions of *P. argentipes* at a micro-scale level under an IRS-intervention condition.

Our results demonstrated that household-based acceptability of SP-IRS was higher in all HTs and most of the households were fully sprayed. Bioassay results showed that *P. argentipes* sand flies were highly susceptible to alpha-cypermethrin but manifested a considerably lower susceptibility to DDT in the study villages. The mean mortality rate of *P. argentipes* to DDT below 50% indicates a high-level resistance to DDT. This was in accordance with the results obtained in previous studies conducted in different time at different villages in Indian VL-endemic states, including Bihar [8, 9, 39, 40]. Along with insecticide susceptibility, the residual efficacy and the intervention effect of insecticides are crucial information. The duration of the residual effect is important for programming cycles. It determines gaps between IRS-rounds so that the population remains protected until the next spraying is performed. Cone bioassay results revealed a considerable variation in mortality rates between wall-surface types at different time intervals post-IRS. Mortality rates on DDT-sprayed surfaces always recorded below the WHO-satisfactory level (i.e. ≥ 80%); while on the SP-sprayed walls, the mortality rate was found to maintain the satisfactory level up to the fourth week post-IRS. From these results, it is clear that although the local *P. argentipes* in the study area are highly susceptible to SP, the residual efficacy of SP varies between HTs. Similar to DDT, SP also failed to achieve the duration of effectiveness reported in the WHO recommendation [41, 42]. This ineffectiveness could be due to improper implementation of IRS (i.e. moving pump at the appropriate speed, distance from the wall, discharge rate and the size of water droplets and its deposition on the wall), and injudicious use of insecticide (i.e. solution preparation) [11, 28, 43]. However, since the present study was conducted under intense supervision and monitoring activities, an alternative reason for not achieving the WHO recommended effectiveness period could be the quality of the SP (i.e. the percentage of the active ingredient or ‘ai’), which accounts for quality control.

Among the three surface types evaluated for insecticide persistence, a significant difference in mortality rates was observed between BUU and CPLC for both insecticides. A further novel finding is that the CPLC recorded better residual efficacy followed by the BUU and the PMP surfaces at almost all intervals post-spraying. However, PMP recorded the highest and second-highest mortality rates for DDT and SP, respectively, at two weeks post-IRS. This result indicates that the maintenance of insecticide deposition on PMP surfaces did not last for a long duration. Such variation in the residual efficacy of insecticides between wall types may have several causes, such as composition of walls chemicals (that increase the pH, causing rapid breakdown of some insecticides), absorbance rate (higher in soil wall), availability of degrading bacteria and degradation rate of wall surface materials, and temperature and humidity [44–49]. Our findings support the results of several other studies which dealt with the residual efficacy of insecticide-treated surfaces against the vectors of different diseases [45, 46, 50, 51].

Estimation of *P. argentipes* reduction rates in the sprayed households showed that the SP-IRS had a better intervention effect on sand flies than DDT-IRS (*P* < 0.001) for all interval periods post-IRS. Reduction rates in the sprayed households between two and 12 weeks ranged between 55.6–90.5% and 14.1–34.1% for SP-IRS and DDT-IRS rounds, respectively. These results also showed a noticeable effect on *P. argentipes* abundance in the sentinel households up to four weeks post-IRS; at 12 weeks post-IRS, *P. argentipes* counts were increased for both IRS-rounds. However, the sand fly counts in the sentinel households between the two IRS rounds showed no significant difference (*P *= 0.33). The results of statistical analysis of *P. argentipes* densities between household groups in each round also revealed a non-significant difference for all four household groups (i.e. sprayed *vs* sentinel; sprayed *vs* control; sentinel *vs* control; and fully *vs* partially) for DDT-IRS and two household groups for SP-IRS (i.e. sentinel *vs* control; and fully *vs* partially). However, *P. argentipes* densities in partially- and fully sprayed households were observed to vary significantly between DDT- and SP-IRS-rounds. This observation, coupled with the fact that the intervention effect was calculated multiple times post-IRS suggests that SP is effective for sand fly control in the households that were either partially or fully sprayed rather than unsprayed. However, although a non-significant statistical difference in sand fly count in sentinel households was observed between DDT-IRS and SP IRS-rounds, mean sand flies collected during the DDT-IRS round had outnumbered compared to SP-IRS round. This result indicates that the insecticide with the highest susceptibility of a vector with a maximum IRS-coverage in a household group can create a mass effect for sand fly control in the unsprayed households. Based on the results, SP provides a better prevention effect to sand fly bites in comparison to DDT in the days following IRS. Furthermore, alpha-cypermethrin belongs to the SP group, which is a contact irritant and immediately toxic to sand flies, is suitable for IRS [51, 52]. This could be one of the primary reasons for the minimal effect of alpha-cypermetrin in the sentinel houses. In another study [52], it was demonstrated that although alpha-cypermethrin exhibited existing response and high knockdown in laboratory assays and inside huts, this compound did not elicit a repellent response from mosquitoes under controlled laboratory conditions or repel mosquitoes from entering huts in the field.

In the present study, three types of spatial risk-maps were developed; household level risk scores and spatial risk-zones are evaluated with the field observations of *P. argentipes* densities. HT-based risk-zone analysis explored that most of the village area (> 78%) in Lawapur Mahanar were covered under the highest level of sand fly emergence and resurgence risks. This could be the main reason for the highly endemic burden of VL in Lawapur Mahanar. The general ISV & IRSS and the final combined risk-maps both had found to produce a low percent of area under the high-risk zone during SP-IRS round rather than the DDT-IRS round. After SP-IRS, a large area from the high and medium HT-based risk-zones was converted into a low-risk zone (i.e. 60.5%; estimated through the combined risk map), which is estimated almost four times lower (16.2%) than in the DDT-IRS combined risk map. This result indicates that IRS is the right choice for sand fly control; however, the degree of protection is based on quality, susceptibility (to the targeted vector), acceptability (during IRS) and implementation of the insecticides.

Results of the household risk score evaluation revealed a good agreement (*P* < 0.05) between the estimated risk-scores and the *P. argentipes* densities collected from the different households. This indicates that identified household-risk parameters and its categorical risk scores were highly suitable for estimating local *P. argentipes* abundance. The *R*^2^ values of agreement analysis for DDT post-IRS weeks were ≥ 0.78, which are equal to or higher than the pre-IRS value (i.e. 0.78). The result indicates an efficacious impact of DDT-IRS on all HTs risk-zones (i.e. high, medium and low). While for the SP-IRS round, *R*^2^ values were found to fluctuate at the second- and fourth-week intervals post-IRS; values at the two-week pre- and at the 12-week post-IRS intervals were almost the same. This result reflects a striking intervention effect of SP-IRS on sand flies with a decreasing trend over time intervals post-IRS. The impact of SP-IRS is highlighted and discussed well in the previous sections.

Field validation results of the combined map risk-zones showed that the highest number (i.e.> 55%) of *P. argentipes* were collected from the high-risk zone followed by medium and low-risk zones for both the IRS-rounds. Hence, GIS-based spatial-risk assessment proves to be an efficient decision-making tool for sand fly risk-zones identification in a separate or combined way of aggregating the various spatial data layers. The developed risk-map provides a complete knowledge of the study area’s pre- and post-intervention conditions (i.e. household-types, IRS-status and intervention effects) that need to be undertaken or improved immediately, especially at a micro-scale level in a highly endemic situation. In fact, there are several studies where GIS-tools have been used for risk-mapping of vectors’ breeding sites and diseases’ spatial distributions at a macro-scale level [24, 26, 37].

The housing characteristics and the IRS intervention-based risk-factors used for *P. argentipes* density analysis are statistically evaluated. Although all six factors (i.e. TF, TW, TR, DS, ISV and IRSS) are significantly associated with local *P. argentipes* abundance in the course of univariate analysis, only five of them were selected in the final multiple regression model. The result showed that the housing characteristics and the IRS-intervention factors such as TF, TW, DS, ISV and IRSS are suitable for monitoring emergence and resurgence, and the propagation of *P. argentipes* in the study area. TR was found non-significant in the multiple regression analysis and thus not selected in the final model. The final model is highly significant and showed that the selected parameters could explain 89% of the *P. argentipes* density. The result of model accuracy assessment showed a robust correlation between predicted and observed *P. argentipes* densities. Our results also support the findings of earlier studies discussing socioeconomic and housing risk-factors in relating to VL-endemicity and spatial distribution of vector in rural Bihar [15, 29].

In the present study, we did not evaluate the insecticides’ deposition on sprayed walls and the quality (i.e. % of ai) of insecticide used for IRS. Deviation in quality and quantity of the insecticides will affect the sand fly mortality rates and intervention effect of the IRS. Thus, estimated mortality rates between surface-types and the intervention effects between household groups may vary from the actual results. A new study may be planned, considering these points. The total risk area estimated for the study village (through GIS risk mapping) includes a vacant area between household hamlets; which had influenced the risk zone levelling (i.e. area identification) and are extension under the different risk zones. However, this study is performed at a micro-scale level; hence, the effect of vacant land on risk zone classification is minimal. Moreover, identification and estimation of different risk-zones on the total village area could provide an alternative for area selection (especially to choose the low risk-areas) for the construction of new houses in the future. Overall, the results of this study provide varying information which was previously unexplored at a micro-scale level. Most importantly, a spatial view of the village risk-map helps to identify and group the households under different risk-zones; this is easy, convenient, cost-effective, less time-consuming than conventional ground survey, and provides information for decision makers.

## Conclusions

Our results conclude that local *P. argentipes* in the study village have developed resistance (i.e. highly resistant) to DDT; sand fly resurgence and emergence observed immediately after IRS. Alpha-cypermethrin seems to be the right choice for IRS for VL-vector control for its 100% mortality and better intervention effect against *P. argentipes*, and for its better community acceptance compared to DDT-IRS. Nevertheless, sand fly mortality rates on SP-sprayed walls were found to vary between surface-types; an ineffective residual efficacy was observed, and it failed to achieve the WHO-recommended time post-IRS. This study provides a good starting point for discussion, and the result warrants further investigation to reveal actual underlying causes. The predictive accuracy of the sand fly density analytical model demonstrates that housing characteristics, insecticide susceptibility of the vector and IRS-status combination could be useful in evaluating *P. argentipes* density in VL-endemic villages of Bihar. Our study also demonstrates that the GIS-based combined spatial-risk mapping (at a macro level) can be a useful tool for risk-zone identification for monitoring the emergence and resurgence of sand files in pre- and post-IRS sessions. Furthermore, the spatial risk map provides a complete knowledge of the extent and nature of different levels of risk-zones, which cannot be explored through conventional field surveys and ordinary data-collection methods. Micro-level spatial-risk information gathered through the GIS-map may help public health scientists and researchers to target different household groups by designing and implementing novel control strategies (i.e. single intervention or integrated vector control) against the nature of risk-levels. Moreover, this risk-map also helps to optimize the allocation and use of control resources in time and places in order to increase programme effectiveness.

## Supplementary information

**Additional file 1: Table S1.** Definition of refused, partially and fully sprayed households in IRS-based VL-vector control programme in Bihar, India. **Table S2.** Weight evaluation and rank based risk-level calibration (for spatial risk-map preparation) of the factors [i.e. household types (HTs), insecticide susceptibility and IRS-status (IS & IRSS)] affecting the emergence and resurgence of *P. argentipes* at a micro-scale level in Vaishali district, Bihar. **Table S3.** Correlation between the household risk scores and the mean *P. argentipes* densities collected in the households in Lawapur Mahanar village of Mahnar block, Vaishali district (Bihar) estimated during DDT- and SP-IRS-rounds in pre- and post-IRS sessions. **Figure S1.** Mean *P. argentipes* densities calculated in sprayed, sentinel and control households at time points pre-IRS (2 weeks) and 2, 4 and 12 weeks post-IRS during DDT- and SP-rounds in Mahnar block, Vaishali district (Bihar). **Figure S2.** Locational distribution of entomological households selected for validating the spatial-risk zones (i.e. low, medium and high levels zones) identified in combined spatial-risk maps (after the IRS interventions using **a** DDT and **b** SP).

## Data Availability

All relevant data are contained within the paper and its additional files.

## Abbreviations

VL: visceral leishmaniasis
IRS: indoor residual spraying
DDT: dichlorodiphenyltrichloroethane
GIS: geographical information system
GPS: global positioning system
SP: synthetic pyrethroid
PMP: plain mud plastered
CPLC: cement plastered and lime-coated
BUU: brick unplastered and unpainted
TH: thatched
IDW: inverse distance weighting
ISV & IRSSs: insecticide susceptibility of vector and IRS-status
