# Supplementary material for: Assessing the combined effects of household type and insecticide effectiveness for kala-azar vector control using indoor residual spraying: a case study from North Bihar, India
Source: Parasit Vectors. 2019 Aug 22;12:409. doi: 10.1186/s13071-019-3670-x (PMC6705094; doi:10.1186/s13071-019-3670-x)

**Additional file 1: Table S1.** Definition of refused, partially, and fully sprayed houses in IRS-based VL-vector control programme in Bihar, India.

| Word detail | Definition |
| --- | --- |
| Refused houses | Houses in an intervention village those owners refused permission to IRS. |
| Partially sprayed house | A house in an intervention village where spraying is not done in all rooms (one or, more rooms remained unsprayed including kitchen, toilet, and animal shelters) or room walls are not covered up to the 6feet height. |
| Fully sprayed house | A house in an intervention village where IRS is done up to the 6feet height in all walls of the rooms including kitchen, toilet, and animal shelters. |

**Table S2.** Weight evaluation, and rank based risk-level calibration (for spatial risk-map preparation) of the factors [i.e. household types (HTs); insecticide susceptibility and IRS-status (IS&IRSS)] affecting the emergence and resurgence of *P. argentipes* at a micro scale level in Vaishali district, Bihar.

| Risk-maps | Factors | Weight (%) | SFRI class (%) | Rank | Degree of risk |
| --- | --- | --- | --- | --- | --- |
| Risk-map DDT-IRS | Household types (HTs) | 50 | < 25 | 1 | Low |
|  |  |  | 25 - 50 | 2 | Medium |
|  |  |  | > 50 | 3 | High |
|  | Insecticide susceptibility and IRS-status (IS&IRSS) | 50 | < 50 | 1 | Low |
|  |  |  | 50.1 - 75 | 2 | Medium |
|  |  |  | > 75 | 3 | High |
| Risk-map SP-IRS | Household types (HTs) | 50 | < 25 | 1 | Low |
|  |  |  | 25 - 50 | 2 | Medium |
|  |  |  | > 50 | 3 | High |
|  | Insecticide susceptibility and IRS-status (IS&IRSS) | 50 | 0 | 0 | No/Low |
|  |  |  | 01 - 50 | 1 | Medium |
|  |  |  | > 50 | 2 | High |

**Table S3.** Correlation between the household risk scores and the mean *P. argentipes* densities collected in the households in Lawapur Mahanar village of Mahnar block, Vaishali district (Bihar) estimated during DDT- and SP-IRS-rounds in pre- and post-IRS sessions.

| Insecticides | IRS-session | Weeks | Observations (n) | Multiple R | R square | *t stat* | *P-value* |
| --- | --- | --- | --- | --- | --- | --- | --- |
| DDT | Pre-IRS | 2 | 12 | 0.88 | 0.78 | 5.95 | 0.00 |
|  | Post-IRS | 2 |  | 0.90 | 0.81 | 6.63 | 0.00 |
|  |  | 4 |  | 0.88 | 0.78 | 5.98 | 0.00 |
|  |  | 12 |  | 0.91 | 0.83 | 7.02 | 0.00 |
|  |  | Overall |  | 0.92 | 0.85 | 7.60 | 0.00 |
| SP | Pre-IRS | 2 |  | 0.91 | 0.82 | 6.73 | 0.00 |
|  | Post-IRS | 2 |  | 0.62 | 0.38 | 2.49 | 0.03 |
|  |  | 4 |  | 0.75 | 0.56 | 3.59 | 0.00 |
|  |  | 12 |  | 0.90 | 0.81 | 6.49 | 0.00 |
|  |  | Overall |  | 0.89 | 0.79 | 6.05 | 0.00 |

**Figure S1.** Mean *P. argentipes* densities calculated in sprayed, sentinel and control households at time points pre-IRS (2-weeks) and at 2, 4 and 12-weeks post-IRS during DDT- and SP-rounds in Mahanar block, Vaishali district (Bihar).


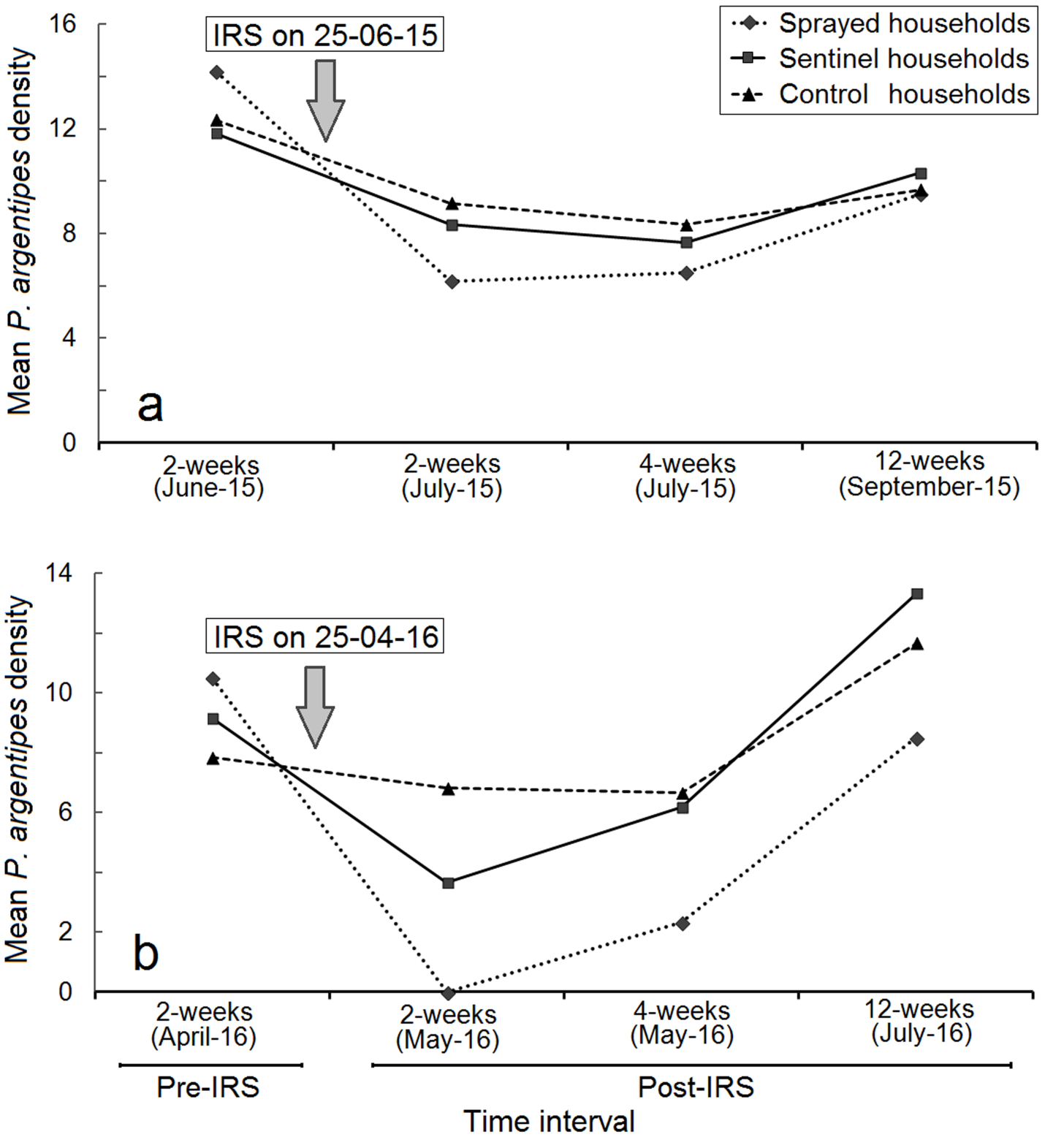


**Figure S2.** Locational distribution of entomological households selected for validating the spatial-risk zones (i.e. low, medium and high levels zones) identified in combined spatial-risk maps [after the IRS interventions using DDT (panel a) and SP (panel b)].


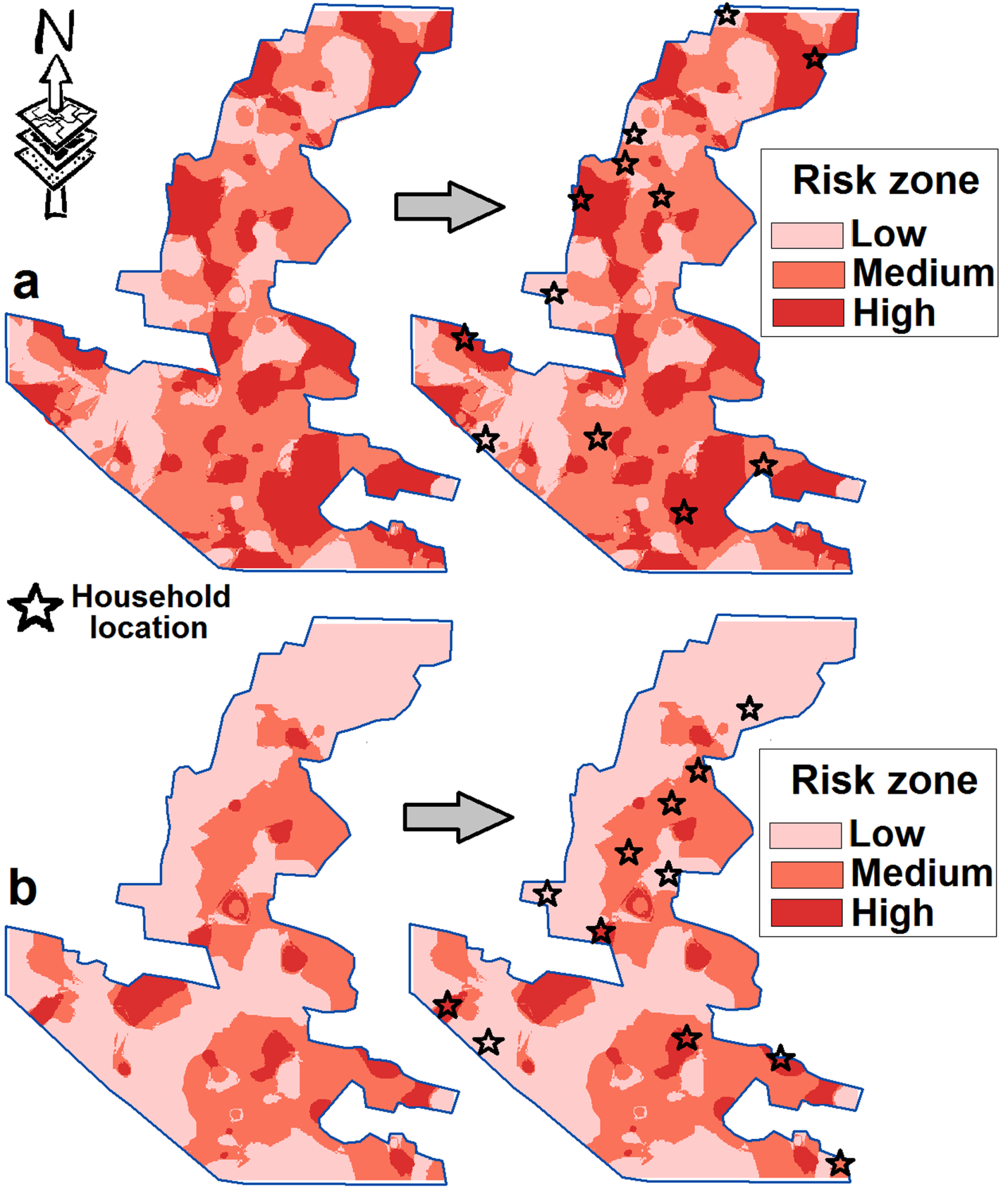

Supplement: Supplementary file 1 — Additional file 1: Table S1. Definition of refused, partially and fully sprayed households in IRS-based VL-vector control programme in Bihar, India. Table S2. Weight evaluation and rank based risk-level calibration (for spatial risk-map preparation) of the factors [i.e. household types (HTs), insecticide susceptibility and IRS-status (IS & IRSS)] affecting the emergence and resurgence of P. argentipes at a micro-scale level in Vaishali district, Bihar. Table S3. Correlation between the household risk scores and the mean P. argentipes densities collected in the households in Lawapur Mahanar village of Mahnar block, Vaishali district (Bihar) estimated during DDT- and SP-IRS-rounds in pre- and post-IRS sessions. Figure S1. Mean P. argentipes densities calculated in sprayed, sentinel and control households at time points pre-IRS (2 weeks) and 2, 4 and 12 weeks post-IRS during DDT- and SP-rounds in Mahnar block, Vaishali district (Bihar). Figure S2. Locational distribution of entomological households selected for validating the spatial-risk zones (i.e. low, medium and high levels zones) identified in combined spatial-risk maps (after the IRS interventions using a DDT and b SP). [file 13071_2019_3670_MOESM1_ESM.docx]
